# Cluster randomized controlled trial of a mobile market intervention to increase fruit and vegetable intake among adults in lower-income communities in North Carolina

**DOI:** 10.1186/s12966-017-0637-1

**Published:** 2018-01-05

**Authors:** Lucia A. Leone, Gina L. Tripicchio, Lindsey Haynes-Maslow, Jared McGuirt, Jacqueline S. Grady Smith, Janelle Armstrong-Brown, Ziya Gizlice, Alice Ammerman

**Affiliations:** 1 Department of Community Health and Health Behavior, University at Buffalo, Buffalo, NY USA; 2 Center for Health Promotion and Disease Prevention, University of North Carolina at Chapel Hill, Chapel Hill, NC USA; 3 Department of Agricultural and Human Sciences, North Carolina State University, Raleigh, NC USA; 4 RTI International, Research Triangle Park, NC USA

**Keywords:** Food environment, Mobile market, Lower-income, Cluster-randomized trial, Fruits and vegetables

## Abstract

**Background:**

Poorer diets and subsequent higher rates of chronic disease among lower-income individuals may be partially attributed to reduced access to fresh fruits and vegetables (F&V) and other healthy foods. Mobile markets are an increasingly popular method for providing access to F&V in underserved communities, but evaluation efforts are limited. The purpose of this study was to determine the impact of Veggie Van (VV), a mobile produce market, on F&V intake in lower-income communities using a group randomized controlled trial.

**Methods:**

VV is a mobile produce market that sells reduced-cost locally grown produce and offers nutrition and cooking education. We recruited 12 sites in lower-income communities in North Carolina (USA) to host VV, randomizing them to receive VV immediately (intervention) or after the 6-month study period (delayed intervention control). Participants at each site completed baseline and follow-up surveys including F&V intake, perceived access to fresh F&V and self-efficacy for purchasing, preparing and eating F&V. We used multiple linear regression to calculate adjusted differences in outcomes while controlling for baseline values, education and clustering within site.

**Results:**

Among 142 participants who completed the follow-up, baseline F&V intake was 3.48 cups/day for control and 3.33 for intervention. At follow-up, adjusted change in F&V consumption was 0.95 cups/day greater for intervention participants (*p* = 0.005), but was attenuated to 0.51 cups per day (*p* = 0.11) after removing extreme values. VV customers increased their F&V consumption by 0.41 cups/day (*n* = 30) compared to a 0.25 cups/day decrease for 111 non-customers (*p* = 0.04). Intervention participants did not show significant improvements in perceived access to fresh F&V, but increased their self-efficacy for working more F&V into snacks (*p* = 0.02), making up a vegetable dish with what they had on hand (*p* = 0.03), and cooking vegetables in a way that is appealing to their family (*p* = 0.048).

**Conclusions:**

Mobile markets may help improve F&V intake in lower-income communities.

**Trial registration:**

Clinicaltrials.gov ID# NCT03026608 retrospectively registered January 2, 2017.

**Electronic supplementary material:**

The online version of this article (10.1186/s12966-017-0637-1) contains supplementary material, which is available to authorized users.

## Background

In the United States, lower-income Americans have higher rates of chronic disease than those with higher income and are also more likely to have poor diets [1]. Adults who consume fewer foods high in saturated fat, sodium and sugar in favor of fruits and vegetables (F&V) are less likely to develop heart disease, type 2 diabetes, certain types of cancer, and are more likely to sustain a healthy weight [2]. While the benefits of consuming F&V are recognized by many [3], low-income individuals are often unable to purchase as much F&V as they would like due to multiple factors including limited access to high quality, fresh and/or affordable F&V [4, 5]. Thus, strategies to improve access for this population are needed.

While many experts believe that providing more options for purchasing fresh produce in underserved communities could help improve consumption of F&V [6], the research supporting this strategy is limited. Several initiatives have been directed at improving access to fresh produce; for example, the Healthy Food Financing Initiative provides financial incentives for supermarkets to open in communities that have limited food retail options [7]. Despite these efforts, none of the studies examining the effects of new grocery stores have shown an impact on F&V consumption [8–10], although they have shown other benefits regarding improved perceptions of neighborhoods [9]. One challenge faced by larger retail outlets is that in addition to selling fresh produce, they also offer many unhealthy options. Additionally, produce marketed in lower-income communities is sometimes poorer in quality and higher in price [4].

Given the challenges with opening new grocery stores in lower-income areas (e.g., high start-up costs, zoning ordinances, perceived lack of customer demand or buying power), there is a need for alternative food sources; Farmer’s markets, Community-Supported Agriculture (CSA; i.e., commitment to a farm to purchase a share of their produce for a set period of time), and mobile markets generally focus exclusively or predominately on F&V and provide fresh (often locally grown), affordable produce. While these strategies are promising [11–16], there are few studies examining their efficacy and at the time of this study there were no randomized controlled trials. Experts have noted the need for stronger study designs to better understand the relationship between the food environment and diet, and identify scalable solutions [6]. Our study sought to fill this gap by rigorously evaluating the impact of a mobile produce market program on F&V consumption among individuals in lower-income communities in North Carolina.

We conducted a cluster randomized controlled trial to evaluate the impact of Veggie Van (VV), a mobile produce market, on F&V intake of participants living in lower-income communities in NC. This paper reports the effect of 6-months of exposure to the VV program on diet, perceived access to fresh F&V and self-efficacy. We compare effects between intervention and control communities and examine the relationship between VV purchasing and changes in diet.

## Methods

The Green Cart Evaluation Study was an evaluation of the VV program conducted between 2012 and 2015 in four counties in North Carolina. For the evaluation, we randomized 12 potential VV sites to receive the VV program immediately after baseline data collection (intervention) or after a 6-month waitlist period (delayed intervention control). This study was retrospectively registered with Clinicaltrials.gov (ID# NCT03026608) on January 2, 2017.

### Veggie van program

The VV was a mobile produce market, run by the non-profit organization Community Nutrition Partnership, that offered high-quality produce aggregated from multiple local farms to customers at a reduced price [15]. The socioecological model acts as a guiding framework for addressing the complexities associated with dietary intake in high-need populations. The socioecological model posits that multiple levels of influences (e.g., individual, interpersonal, and community-level factors) intersect to yield outcomes [17]. Social Cognitive Theory provides constructs that support the goal of targeting behavior change on at the individual and environmental levels. Specifically, VV sought to change the food environment (and people’s perceptions of it) while simultaneously improving self-efficacy to purchase, prepare and eat fresh F&V.

Aspects of the food environment addressed by VV included availability, accessibility, acceptability, affordability, and accommodation [18]. VV increased the number of food outlets within target communities by partnering with local community organizations that were already frequented by or in locations near the target population (accessibility) and selling produce at those locations (availability). Locations included health clinics, recreation centers, libraries, housing communities and community centers [15, 19]. The VV mobile market was held weekly during the 6-month intervention period, unless staffing or weather issues prevented the market from operating. Customers could pay week-to-week for shares of produce (i.e., similar to a CSA they would receive a set amount of seasonally available produce items offered at the same price each week) or buy individual produce items at the market. In order to ensure VV offered high quality produce, all the F&V were fresh, locally grown, and often organic (acceptability). Produce was offered at about half the cost of traditional CSA programs (affordability). Unlike CSA programs, no upfront payments or commitments were required though pre-ordering was encouraged and incentivized with monetary discounts. To maximize accommodation, VV accepted multiple forms of payment (cash, credit/debit, check and the Supplemental Nutrition Assistance Program’s (SNAP) electronic benefit transfer (EBT) cards) and visited sites at times which were assessed to be the most convenient for potential customers.

The educational intervention addressed individual skills and behaviors related to F&V consumption with the goal of improving self-efficacy through increased outcome expectations/expectancies, observational learning and behavioral capability. Newsletters and nutrition demonstrations addressed the benefits of healthy eating (expectations). Social marketing campaigns at VV sites encouraged people to come to markets to benefit from VV (expectancies). VV also provided cooking demonstrations, tips for cooking seasonal produce and recipes for items sold at VV (behavioral capability and observational learning). While outside the context of research, customers only received weekly newsletters when they visited the VV, for the purposes of this evaluation, research participants at intervention sites received newsletters by mail or e-mail even if they did not visit VV. Since we could not require study participants to shop at VV, we sent them the newsletters as a way of ensuring that they were aware of the program and at least received some dose of the behavioral intervention.

### Recruitment

The Green Cart study and VV teams partnered with organizations that were serving the priority population (lower-income and/or limited access to fresh produce) to facilitate the research. Site and participant recruitment are described in detail elsewhere [19, 20]. We asked partner organizations to collect at least 30 interest forms from community members who were potentially interested in purchasing VV produce and who were willing to participate in a research study. A member of the research team contacted those who completed the forms and asked them to participate in a study. Eligible individuals (Age 18 or older, English speaking, and primary food shopper for their household) were invited to complete a telephone-administered baseline survey and were enrolled in the study. After participant data collection was completed, we randomized sites in pairs to either the intervention or the delayed intervention control group. Institutional Review Board at the university approved all procedures.

### Survey and measures

We collected all data over the phone via interviewer-administered surveys at baseline and 6-months. Additional details on data collection and measures can be found elsewhere [20].

### Dietary outcomes

The primary outcome, F&V intake (cups/day), was assessed using the 10-item National Cancer Institute F&V screener and calculated according to the screener instructions [21]. A validation study found estimated correlations between the screener and F&V intake from 4 non-consecutive 24-h recalls were 0.67 for men and 0.53 for women [22]. Test-retest reliability for similar food frequency questionnaires (FFQs) is generally good (ICC = 0.65) [23]. Added sugar (servings/day) was calculated from the 7-items selected from the National Health and Nutrition Examination Survey Dietary Screener to capture consumption in the past month including sugar-sweetened beverages, chocolate or candy, pastries, desserts or ice cream [24].

### Psychosocial measures

In order to measure the impact of the environmental-level intervention components, we assessed participant’s perceived access to fresh F&V assessed using a 3-item scale [25, 26] adapted to examine access using three different definitions: one assessing perceived neighborhood access, one assessing perceived access near the VV location in their community, and one assessing general perceived access. Possible perceived access scores range from 3 (strongly disagree to all items) to 15 (strongly agree to all items), with a midpoint of 8 indicating a neutral response. The impact of the nutrition education intervention component was assessed by looking at self-efficacy to purchase, prepare and eat F&V. Self-efficacy was assessed using nine questions (shown in Table 4) with response options ranging from 1 to 10 (1 = “very easy” to 10 = “very hard”) [27]. Items were summed to create a total self-efficacy score (range 9 to 90).

### VV usage and implementation measures

On the survey, participants were asked to report if they had ever used VV; we also reviewed sales data to see if participants made any purchases at VV during the intervention period. If a participant purchased at least one share of produce, they were recorded as a customer. Participants who only purchased individual produce items were not identified by name and not included in the sales data. In order to understand VV program implementation and fidelity, coordinators were asked to fill out a process measures form after each market. Process data will be reported separately.

### Power analysis

Our original power analysis was based on F&V servings/day as calculated by the National Cancer Institute F&V screener. In the sample size estimates, we considered correlated change in F&V intake among participants within a community site (ICC), number of participants within each site and number of sites (clusters) [28, 29]. The VV program was expected to increase the F&V consumption by least 1.25 servings per day or approximately 0.75 cups/day (effect size of approximately 0.35) based on the VV pilot [15]. A sample size of 6 communities per group with 20 participants in each community yielded 0.80 power to detect 0.75 cup difference in mean changes between two groups using two-sided tests of significance at *p* = 0.05, assuming an ICC of 0.001 and standard deviation of 3.6 based on other cluster randomized trials [30]. We assumed attrition to be no more than 20% based on the pilot study [15]. Thus, a final goal was to recruit at least 25 participants in each community, for a total of 300 participants.

### Analysis

A generalized linear mixed model (GLMM) with a random intercept to control for clustering within community sites was used to test the effect of the VV intervention on dietary intake (F&V and added sugar) at 6-months. Additional GLMM variables included: 1) baseline diet as fixed covariate, and 2) baseline diet and education. Including the baseline values as a covariate, in an analysis of covariance (ANCOVA) is known to be a more powerful test than a group comparison of baseline to post-intervention change (26). ANCOVA is not distorted by regression towards the mean bias, whereas a change analysis is subject to that bias [31, 32]. We controlled for education because both income and education were significantly higher among control participants at baseline. These items were highly correlated and education was more consistently reported than income. Additionally, we completed a sensitivity analysis on the final model excluding extreme F&V reporters. Extreme F&V reporters were defined as participants who had a change greater than 9-cups of F&V per day. All secondary outcome analyses were conducted using GLMMs (e.g., usage, perceived access, self-efficacy), removed extreme reporters (when F&V intake was the outcome) and controlled for baseline values, education and clustering within sites.

## Results

**Fig. 1:**
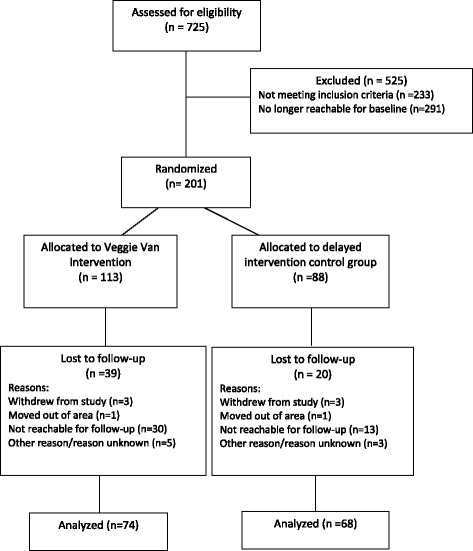
Recruitment and randomization of participants for the Veggie Van mobile market program and the Green Cart Evaluation study

Interest forms were collected from 516 individuals across 12 sites. A total of 201 participants enrolled into the study and completed baseline measures: 113 intervention and 88 control. Site-by-site recruitment ranged from 23.5% to 50% (see Additional file 1). A total of 142 participants completed the 6-month follow-up survey: 74 intervention and 68 control; this represented a 70.6% retention rate. Site-by-site retention ranged from 39.1% to 90% (see Additional file 1). Study participant drop-out was predominately due to the research team not being able to contact the participant (e.g., participant was non-responsive after 6 or more attempts, number was disconnected, and alternate contact attempts were not successful) (Fig. 1). There were no differences between study completers and drop-outs in regards to any of the demographic data or F&V consumption at baseline.

### Demographics

Baseline characteristics for 142 study completers, by treatment arm, are summarized in Table 1. Overall, the sample was predominately female (95.8%), African American (64.8%), and not married (66.7%). On average, the sample was 46.3 ± 14.0 years old, obese (mean BMI = 31.3 ± 7.8), and had two adults and two children living in the household. More than half (54.0%) made less than $30,000 per year and 62.0% received some form of government assistance (i.e., SNAP, Medicaid).

**Table 1:** Baseline characteristics of Veggie Van study Participants by intervention condition

Variable	Entire Sample	Intervention	Control
Gender, %			
*Male*	4.2%	6.8%	1.5%
*Female*	95.8%	93.2%	98.5%
Age, mean (SD)	46.30 (14.01)	47.89 (14.89)	44.52(12.83)
Number of Adults in Household, mean (SD)	1.96 (1.17)	1.99 (1.45)	1.93 (0.78)
Number of Children in Household, mean (SD)	2.17 (1.24)	2.22 (1.40)	2.12 (1.04)
Hispanic/Latino, %	2.1%	2.7%	1.5%
Race, %			
*Black/African American*	64.8%	68.1%	64.2%
*White*	31.0%	29.2%	34.3%
*American Indian*	0.7%	1.4%	0%
*Other*	1.4%	1.4%	1.5%
Marital Status, %			
*Married*	33.3%	30.1%	36.8%
*Never Married*	46.8%	47.9%	45.6%
*Divorced/Separated/Widowed*	19.9%	21.9%	17.6%
Education, %			
*High School Grad or less, GED, Trade or Beauty School*	32.9%	37.8%	27.3%
*Some College*	26.4%	25.7%	54.5%
*College Graduate*	20.7%	17.6%	24.2%
*More than college*	20.0%	18.9%	21.2%
Income, %			
*< 10,000*	21.8%	28.6%	14.8%
*10,000-29,999*	32.2%	34.9%	29.6%
*30,000-49,999*	21.0%	14.2%	27.9%
*50,000 or more*	25.0%	22.2%	27.9%
BMI, mean (SD)	31.29 (7.81)	31.14 (7.85)	31.46 (7.82)
Receiving Government Assistance, %			
*SNAP*	33.8%	29.7%	38.2%
*WIC*	19.0%	16.2%	22.1%
*Free/reduced price lunch*	30.3%	31.1%	29.4%
*Medicaid*	43.7%	48.6%	38.2%
*TANF*	3.5%	5.4%	1.5%
*None*	38.0%	35.1%	41.2%

*N* = 142 (Intervention *n* = 74; Control *n* = 68) for measures of gender, number of adults in the household, number of children in the household, Hispanic/Latino; *N* = 140 (Intervention *n* = 74; Control *n* = 66) for age and education; *N* = 139 (Intervention *n* = 72; Control *n* = 67) for race; *N* = 141 (Intervention *n* = 73; Control *n* = 67) for marital status; *N* = 124 (Intervention *n* = 63; Control *n* = 61) for income; *N* = 131 (Intervention *n* = 70; Control *n* = 61) for BMI

*SD* standard deviation, *GED* general educational development proficiency, *BMI* body mass index, *SNAP* supplemental nutrition assistance program, *WIC* Special Supplemental Nutrition Program for Women, Infants and Children, *TANF* Temporary Assistance for Needy Families

### Dietary intake

#### Intervention vs. control

Dietary intake data are shown in Table 2. F&V intake at baseline was 3.33 (SD 2.36) cups/day for the intervention group and 3.48 (SD 3.14) cups/day for the control group. At follow-up, intervention participants increased their intake by 0.31 cups/day and control participants decreased their intake by 0.66 cups/day. Intervention participants had significantly higher F&V intake than control participants at follow-up (*p* = 0.05). The difference in mean changes after adjusting for clustering, baseline intake and education was 0.95 cups per day (*p* = 0.005). We conducted a sensitivity analysis removing four participants who had extreme F&V changes and found that the difference was attenuated to 0.51 cups/day (*p* = 0.11). The intraclass correlation (ICC) for change in F&V intake by site was 0.08.

**Table 2:** Changes in dietary intake among Veggie Van study completers by intervention condition

F&V Intake (Cups/Day)				
Outcome	Intervention (*n* = 74) *Mean† (SE)*	Control (*n* = 68) *Mean† (SE)*	Intervention Effect *Mean Difference† (SE)*	*P*-value†
Baseline	3.33 (0.27)	3.48 (0.38)	−0.15 (0.46)	0.75
6-month Follow-up	3.64 (0.30)	2.82 (0.19)	0.81 (0.36)	0.05*
Change at 6-months controlling for clustering				
	0.41 (0.46)	−0.64 (0.46)	01.06 (0.65)	0.14
Change at 6-months controlling for clustering and baseline F&V intake				
	0.34 (0.31)	−0.59 (0.32)	0.93 (0.45)	0.06
Change at 6-months controlling for clustering, baseline F&V intake, and education				
	0.30 (0.28)	−0.65 (0.09)	0.95 (0.30)	0.005*
Change at 6-months controlling for clustering, baseline F&V intake, and education (extremes removed)				
	0.14 (0.20)	−0.37 (0.20)	0.51 (0.28)	0.11
Added Sugar Intake (Servings/Day)				
Outcome	Intervention *Mean† (SE)*	Control *Mean† (SE)*	Intervention Effect *Mean Difference† (SE)*	*p*-value
Baseline	2.31 (0.26)	1.84 (0.18)	0.47 (0.31)	0.14
6-month Follow-up	2.19 (0.23)	1.71 (0.16)	0.48 (0.32)	0.10
Change at 6-months controlling for clustering				
	−0.15 (0.27)	−0.13 (0.27)	0.02 (0.38)	0.96
Change at 6-months controlling for clustering and baseline sugar intake				
	−.07 (0.23)	−.26 (0.23)	0.19 (0.32)	0.51
Change at 6-months controlling for clustering, baseline sugar intake, and education				
	−.07 (0.23)	−.26 (0.23)	0.19 (0.32)	0.52

†All means, mean differences and *p*-values are adjusted for clustering within sites and any other controls specified within the model; *statistically significant (*p* < 0.05)

*F&V* fruit and vegetables, *SD* Standard Deviation, *SE* Standard Error

Sugar intake at baseline was 2.31 (SD 2.23) servings/day for the intervention group and 1.84 (SD 1.45) servings/day for the control group. Both groups decreased their sugar intake at follow-up, but there were no significant changes in added sugar intake between the intervention and control groups at 6-month follow-up in any of the models.

#### F&V intake by veggie van usage

At 6-month follow-up, almost all intervention participants reported being aware of the VV at their site (*n* = 71, 95.5%). Of those, 47 intervention participants self-reported ever purchasing from VV (63.5%). Additionally, 2 control participants also reported shopping at VV during the initial 6-month period. We compared change in F&V intake for those who reported ever purchasing from VV (+0.07 cups, *n* = 47 after two removing extreme F&V reporters) versus those who did not report shopping at VV (−0.26 cups/day, *n* = 85) and did not find any statistically significant differences (*p* = 0.32). However, VV sales data indicated that participants who had at least one share purchase on record during the intervention period reported greater adjusted changes (0.41 cups/day, *n* = 30) in F&V consumption at 6-months than those who had no purchasing record (−0.25 cups/day, *n* = 111); this represented a 0.67 cup/day difference overall between groups (*p* = 0.04). There was also some evidence of dose-response among those who used VV; after adjusting for co-variates intervention participants who reported purchasing from VV three or four times over the past month (*n* = 8) had a 1.14/cup per day greater change in F&V intake compared to those who reported 1 or 2 purchases over the past month (*n* = 23): +0.47 cups/day vs. -0.67 cups/day (*p* = 0.04).

### Perceived access

Perceived access to fresh F&V is reported in Table 3. There were no statistically significant changes in neighborhood perceived access, perceived access near the VV site, or general perceived access at 6-months. We also did not observe any changes in participants reporting that they could afford enough F&V to feed their families.

**Table 3:** Change in perceived access to fresh fruits and vegetables for study completers by intervention condition

Perceived Access Scale	Intervention	Control	Intervention Effect *Adjusted Mean Difference* ^*a*^ *(SE), p-value*
General (3-item)			
*Baseline, mean (SD)*	11.12 (3.11)	10.72 (2.65)	−0.23 (0.37)
*Adjusted Change*^*a*^*, mean (SE)*	0.74 (0.27)	0.97 (0.27)	*p* = 0.54
Veggie Van Site (3-item)			
*Baseline, mean (SD)*	8.10 (3.40)	8.34(2.96)	0.46 (0.69)
*Adjusted Change*^*a*^*, mean (SE)*	1.57 (0.49)	1.10 (0.49)	*p* = 0.52
Home (3-item)			
*Baseline, mean (SD)*	10.38 (3.33)	9.93 (3.28)	−0.38 (0.64)
*Adjusted Change*^*a*^*, mean (SE)*	0.26 (0.45)	0.64 (0.46)	*p* = 0.57
Afford F&V (1-item)			
*Baseline, mean (SD)*	3.46 (1.21)	3.24 (1.22)	0.07 (0.17)
*Adjusted Change*^*a*^*, mean (SE)*	0.23 (0.12)	0.16 (0.12)	*p* = 0.67

^a^Analysis adjusted for site, baseline perceived access and education

N = 142 for general scale and Afford F&V (intervention = 74, control = 68); N = 131 for Veggie Van Site scale (intervention = 167, control = 64); N = 139 for home scale (intervention = 72, control = 67)

*F&V* Fruits and Vegetables, *SD* Standard Deviation, *SE* Standard Error

### Self-efficacy

Self-efficacy at 6-months is reported in Table 4. While total self-efficacy at follow-up was slightly higher for the intervention group (61.5) vs. the control group (57.4), this difference was not statistically significant (*p* = 0.10). Intervention participants did have greater improvements in self-efficacy compared to control for three scale items: working more F&V into snacks (1.23, *p* = 0.02), making up a vegetable dish with what they had on hand (0.94, *p* = 0.03), and cooking vegetables in a way that is appealing to their family (0.89, *p* = 0.048).

**Table 4:** Change in self-efficacy for purchasing, eating and preparing fruits and vegetables by intervention condition

Self-Efficacy Item		Intervention (*n* = 64)	Control (*n* = 64)	Intervention Effect *Adjusted Mean Difference† (SE),* *p-value*
1. How easy or hard would it be for you to buy more fruits and vegetables than you normally do the next time you shop?	*Baseline, mean (SD)*	6.74 (3.24)	6.90 (3.01)	0.31 (0.44)
	*Adjusted Change†, mean (SE)*	0.71 (0.31)	0.40 (0.32)	*p* = 0.49
2. How easy or hard would it be for you to use all of the fruits and vegetables that you buy before they go bad?	*Baseline, mean (SD)*	7.35 (2.73)	6.83 (2.71)	−0.01 (0.40)
	*Adjusted Change†, mean (SE)*	0.46 (0.29)	0.47 (0.29)	*p* = 0.98
3. How easy or hard would it be for you to work more fruits and vegetables than you normally do into meals for yourself and your family?	*Baseline, mean (SD)*	7.26 (2.72)	7.29 (2.60)	0.54 (0.42)
	*Adjusted Change†, mean (SE)*	0.46 (0.30)	−0.08 (0.30)	*P* = 0.21
4. How easy or hard would it be for you to work more fruits and vegetables than you normally into snacks for yourself and your family?	*Baseline, mean (SD)*	7.04 (3.05)	7.09 (2.71)	1.23 (0.53)
	*Adjusted Change†, mean (SE)*	1.10 (0.38)	−0.13 (0.38)	*p* = 0.02*
5. How easy or hard would it be for you to cook vegetables in a way that is appealing to your family?	*Baseline, mean (SD)*	7.27 (2.98)	7.67 (2.48)	0.94 (0.42)
	*Adjusted Change†, mean (SE)*	0.89 (0.30)	−0.06 (0.30)	*p* = 0.03*
6. How easy or hard would it be for you to make-up a vegetables dish with what you have on hand?	*Baseline, mean (SD)*	7.55 (2.74)	6.99 (2.77)	0.90 (0.45)
	*Adjusted Change†, mean (SE)*	0.80 (0.32)	−0.10 (0.33)	*p* = 0.048*
7. How easy or hard would it be for you to try vegetables that you have not eaten before?	*Baseline, mean (SD)*	5.75 (3.32)	5.88 (3.14)	0.01 (0.53)
	*Adjusted Change†, mean (SE)*	0.51 (0.37)	0.50 (0.38)	*P* = 0.99
8. How easy or hard would it be for you to prepare and cook new recipes?	*Baseline, mean (SD)*	7.24 (3.03)	6.70 (2.97)	0.39 (0.47)
	*Adjusted Change†, mean (SE)*	0.86 (0.33)	0.46 (0.34)	*p* = 0.40
Self-efficacy Sum	*Baseline, mean (SD)*	55.03 (16.5)	55.17 (14.2)	4.09 (2.45)
	*Adjusted Change†, mean (SE)*	6.40 (1.76)	2.30 (1.73)	*p* = 0.10

† Adjusted analyses control for site, baseline self-efficacy and education; *Statistically significant (p < 0.05)

For self-efficacy sum *N* = 148 (intervention = 64 and control = 64); For intervention group: *n* = 73 for item 1, *n* = 74 for items 2, 6 and 8, *n* = 70 for items 3 and 4, *n* = 71 for items 5 and 7; For control group: n = 68 for items 1, 3, 4 and 5, *n* = 67 for items 2, 5, 7 and 8

*SD* standard deviation, *SE* Standard error

## Discussion

Our findings suggest that participants at sites that received the VV improved their F&V consumption as compared to participants at sites assigned to the control condition, however much of the difference we saw was due to a decrease in the control group and findings were attenuated after extreme values were removed. Our original hypothesis for this research was that in order to accommodate an increase in F&V intake, participants would need to decrease something else in their diet. We expected to see a decrease in sugar as a result, but our data did not confirm this hypothesis.

While this is the first published report of a randomized controlled trial for a mobile market, main outcomes findings are consistent with other pre-post design mobile market studies that generally show an increase in F&V consumption of about 1 cup (2 servings) per day [12–14]. Sub-group analyses looking at F&V consumption among VV shoppers vs. those who did not use VV were similar to previous pilot work that showed a 1.6/serving per day (approx. 0.8 cup/day) improvement for customers compared to non-customers [15]. For the main outcomes analysis, we would expect changes in intake for the current study to be attenuated in comparison to previous studies. Previous research has only looked at changes in intake for those who received the intervention whereas we used an intent-to-treat design and there was no guarantee that the participants recruited for this study would shop at VV. We found that exposure to the environmental intervention was limited; while a majority of intervention participants reported using VV, purchases reported in the past month (when it would likely affect F&V intake at follow-up) were limited. Future studies should consider additional outreach strategies and study designs to ensure that more participants become and remain customers for the duration of the intervention in order to better estimate efficacy. For example, baseline data could be collected at the time of first purchase and subscriptions could encourage continued use. In addition, better sales data are needed to understand dose response; our data were only able to indicate if customers ever purchased a share, but could not track individual produce purchases.

To further understand our findings, we looked at two scales which were meant to measure the effect of two aspects of this intervention as described in our conceptual model [20]. A perceived access scale was designed to capture the environmental aspects of the intervention (improvements in availability, quality, and affordability of fresh produce) and a self-efficacy scale was used to assess behavioral aspects of the intervention (behavioral capability for purchasing, preparing and eating fresh F&V). Perceived access was already high at baseline and while there were small increases in the intervention group, none were statistically significant. This may also be due to the fact that only about 2/3 of participants actually shopped at the VV so not everyone received the full intervention dosage. While nearly everyone in the intervention group was aware of VV, this may not have been enough to increase their perceived access. Thus, we would hypothesize, based on the conceptual model, that the changes we saw in F&V consumption may be partially mediated by changes in self-efficacy due to the behavioral intervention.

Greater F&V consumption for the intervention group and VV users may also be the result of aspects of our mobile market approach that we did not measure directly such as community engagement or the “share” model. Based on qualitative feedback from focus groups with customers (unpublished data), we hypothesize that purchasing a share of produce each week led to more purchasing and consumption than would be seen with a traditional market model of selecting individual produce items. For our model, customers had the option of buying individual pieces of produce or shares. While data indicate that those who ever purchased a share had greater F&V intake at follow-up, purchasing data was not complete enough to look at dose-response. Further research is needed to understand whether sales of shares is associated with greater consumption than would be seen with a traditional market model.

### Limitations

In order to maintain partner engagement, we ended recruitment at most sites before we reached our study goals [19]. Consequently our baseline numbers (*N* = 201) were lower than the anticipated 300. Extended timelines may also have affected drop-out, which was higher than our pilot work (71% actual retention rate vs. 80% anticipated retention rate) [19]. While the research team and the VV staff worked closely together on major decisions that would affect the research, VV staff was ultimately responsible for implementation. Therefore, VV prices, nutrition education programming, and intervention duration were not always consistent across sites. Recruitment and implementation challenges are common in community-based research, especially with minority populations, [33] and the randomized controlled design provides adequate experimental control despite these threats to internal validity.

Another limitation is that this study relied on a food frequency questionnaire (FFQ) to collect diet information. While FFQs are acceptable and have been used in other mobile market research [13, 34], they only reflect limited number of vegetable categories and may not have adequately captured some of the local produce that was commonly available in North Carolina (leafy greens, root vegetables, etc.). As with all self-report dietary assessment measures, results are susceptible to bias. To help improve the accuracy of FFQs, we provided portion size sheets to help participants with their answers, but only about a fifth of participants reported using them when completing the surveys.

As noted above, much of the difference in F&V consumption we saw between the intervention and control groups was due to decreases in the control group. While we don’t know the cause of these decreases, we saw a similar trend in our pilot work which could represent a regression to the mean. We also reviewed the data for a possible effect of seasonality on F&V intake across months and seasons at each time point, but did not find any patterns that suggested a seasonality bias. Future work should consider using 24-h recalls or adapting standard FFQs to account for local or regional produce.

Despite its limitations, these methods represent an improvement to previous mobile market research. Although the study did not reach goal participation numbers, these projections were based on studies that were similar, but that ultimately had different ICCs and standard deviations than the present study. Our estimates also anticipated a smaller (more conservative) difference in the change in F&V intake between intervention and control participants than we saw with our main adjusted analysis so we could more easily detect a significant difference. When extremes removed, the difference was smaller than the anticipated change (0.5/cup vs. a projected 0.75 cups/day difference) which could explain why that comparison did not reach statistical significance.

#### Public health implications

Mobile markets are a promising strategy for improving F&V consumption in lower-income and low food access communities; they have low overhead and start-up costs so they overcome many of the challenges associated with building new food retail. As their sales can be limited to fresh produce and other healthy items, they do not have the same challenges faced by interventions in existing food retail stores, which generally are able to promote healthy items, but are limited in their ability to decrease exposure to unhealthy items. Furthermore, our team designed VV to meet the needs of underserved and lower-income communities in North Carolina. Extensive formative research indicated that mobile markets were needed and wanted in the target communities and were preferred above several possible other strategies [35, 36]. Not only were we able to demonstrate an impact of VV on F&V intake, but the majority of customers also self-reported reported several other benefits to their health and diet.

While VV was able to improve F&V consumption among customers, challenges still exist to implementing this program on a larger scale. First, financial viability is a concern, as many of mobile markets described in the published literature have closed due to issues with the sustainability of the model. VV experimented with a sliding scale model, but found it difficult to sell enough produce on the higher end of the scale to support the reduced cost purchases. Another strategy included reducing waste through pre-ordering of produce, but this can be difficult to manage without sophisticated point-of-sale software. Markets may have success leveraging local programs such as “Double Up Food Bucks” which allow SNAP participants to increase their buying power [37], but need to consider that many lower-income individuals are not eligible for SNAP and may still benefit from reduced cost produce. Future research should aim to better understand approaches for financial sustainability, including looking at creative partnerships with for-profit businesses or cost-offset models.

Mobile market programs also need to consider how to best improve their reach. More focus is needed on community engagement and customer relations as potential mobile market shoppers often have concerns about the quality and affordability of the F&V and the trustworthiness of mobile market providers [36, 38, 39]. The current study sought to improve trust by involving community organizations as liaisons to the VV, however their community outreach capabilities were also limited. Future programs should consider creating community advisory committees or hiring residents as mobile market staff.

## Conclusions

This was the first study to demonstrate using a randomized controlled trial design that mobile produce markets can be an effective means for increasing F&V consumption among lower-income customers. If the recommended improvements to financial models and community engagement can be achieved, VV has the potential to become an effective, scalable intervention for improving F&V consumption in lower-income communities.

## Additional file

Additional file 1: Recruitment and Retention Rates for 12 Community Sites Participating in Veggie Van Study. (DOCX 13 kb)

## Abbreviations

ANCOVA: Analysis of covariance
CSA: Community supported agriculture
EBT: Electronic benefit transfer
F&V: Fruits and vegetables
FFQ: Food frequency questionnaire
GLMM: Generalized linear mixed model
ICC: Intra-class correlation
SNAP: Supplemental nutrition assistance program
VV: Veggie van
