# Supplementary material for: Cluster randomized controlled trial of a mobile market intervention to increase fruit and vegetable intake among adults in lower-income communities in North Carolina
Source: Int J Behav Nutr Phys Act. 2018 Jan 5;15:2. doi: 10.1186/s12966-017-0637-1 (PMC5756418; doi:10.1186/s12966-017-0637-1)
Supplement: Additional file 1: — Recruitment and Retention Rates for 12 Community Sites Participating in Veggie Van Study. (DOCX 13 kb) [file 12966_2017_637_MOESM1_ESM.docx]

| **Site #** | **Condition** | **Completed Interest Form** | **Completed Baseline** | **Completed Follow-up** | **Recruitment Rate^1^** | **Retention Rate^2^** |
| --- | --- | --- | --- | --- | --- | --- |
|  |  |  |  |  |  |  |
| 1 | Control | 65 | 20 | 18 | 30.8% | 90.0% |
| 2 | Intervention | 58 | 29 | 19 | 50.0% | 65.5% |
| 3 | Intervention | 40 | 18 | 12 | 45.0% | 66.7% |
| 4 | Intervention | 50 | 25 | 20 | 50.0% | 80.0% |
| 5 | Control | 33 | 12 | 10 | 36.4% | 83.3% |
| 6 | Control | 39 | 19 | 11 | 48.7% | 57.9% |
| 7 | Control | 53 | 20 | 15 | 37.7% | 75.0% |
| 8 | Intervention | 62 | 23 | 9 | 37.1% | 39.1% |
| 9 | Intervention | 34 | 8 | 5 | 23.5% | 62.5% |
| 10 | Control | 25 | 8 | 7 | 32.0% | 87.5% |
| 11 | Intervention | 33 | 10 | 9 | 30.3% | 90.0% |
| 12 | Control | 24 | 9 | 7 | 37.5% | 77.8% |
| **Total** |  | **516** | **201** | **142** | **39.0%** | **70.6%** |

Recruitment and Retention Rates for 12 Community Sites Participating in Veggie Van Recruitment rate indicates the percentage of potential participants at each site that expressed interest in the Veggie Van program who subsequently completed a baseline survey

^2^Retention rate indicates the percentage of participants (i.e., those who completed a baseline survey) that also completed a 6-month follow-up survey
